# Socioeconomic status has direct impact on asthma control: Turkish adult asthma registry

**DOI:** 10.1002/clt2.70018

**Published:** 2025-01-25

**Authors:** Bahar Arslan, Murat Türk, Serhat Hayme, Ömür Aydin, Derya Gokmen, Gozde Koycu Buhari, Zeynep Celebi Sozener, Bilun Gemicioglu, Ismet Bulut, Sengul Beyaz, Cihan Orcen, Secil Kepil Ozdemir, Metin Keren, Ebru Damadoglu, Tugce Yakut, Ayse Fusun Kalpaklioglu, Ayse Baccioglu, Sumeyra Alan Yalim, Insu Yilmaz, Ilkay Koca Kalkan, Elif Yelda Ozgun Niksarlioglu, Ali Fuat Kalyoncu, Gul Karakaya, Muge Erbay, Sibel Nayci, Fatma Merve Tepetam, Asli Akkor Gelincik, Hulya Dirol, Ozlem Goksel, Selen Karaoglanoglu, Ferda Oner Erkekol, Sacide Rana Isik, Fusun Yildiz, Yasemin Yavuz, Dilek Karadogan, Nurgul Bozkurt, Ummuhan Seker, Ipek Kivilcim Oguzulgen, Ilknur Basyigit, Serap Argun Baris, Elif Yilmazel Ucar, Tuba Erdogan, Mehmet Polatli, Dane Ediger, Fatma Esra Gunaydin, Leyla Pur, Zeynep Yegin Katran, Yonca Sekibag, Enes Furkan Aykac, Dilsad Mungan, Ozcan Gul, Ali Cengiz, Bulent Akkurt, Seyma Ozden, Semra Demir, Derya Unal, Ayse Feyza Aslan, Ali Can, Reyhan Gumusburun, Gulhan Bogatekin, Hatice Serpil Akten, Sinem Inan, Munevver Erdinc, Aliye Candan Ogus, Murat Kavas, Demet Polat Yulug, Mehmet Erdem Cakmak, Saltuk Bugra Kaya, Gulistan Alpagat, Eylem Sercan Ozgur, Oguz Uzun, Sule Tas Gulen, Gulseren Pekbak, Deniz Kizilirmak, Yavuz Havlucu, Halil Donmez, Gulden Pacaci Cetin, Sadan Soyyigit, Bilge Yilmaz Kara, Gulden Pasaoglu Karakis, Adile Berna Dursun, Resat Kendirlinan, Ayse Bilge Ozturk, Can Sevinc, Gokcen Omeroglu Simsek, Oznur Abadoglu, Pamir Cerci, Taskin Yucel, Irfan Yorulmaz, Zahide Ciler Tezcaner, Emel Cadalli Tatar, Ahmet Emre Suslu, Serdar Ozer, Engin Dursun, Arzu Yorgancioglu, Gulfem Elif Celik, Mehmet Atilla Uysal

**Affiliations:** ^1^ Erciyes University School of Medicine, Department of Chest Diseases, Division of Allergy and Immunology Kayseri Turkey; ^2^ University of Health Sciences, Kayseri City Training and Research Hospital Clinic of Allergy and Immunology Kayseri Turkey; ^3^ Erzincan Binali Yıldırım University School of Medicine Department of Biostatistics and Health Informatics Erzincan Turkey; ^4^ Ankara University School of Medicine Department of Chest Disease Division of Immunology and Allergy Ankara Turkey; ^5^ Ankara University Faculty of Medicine Department of Biostatistics Ankara Turkey; ^6^ University of Health Sciences Ankara Ataturk Sanatoryum Training and Research Hospital Department of Immunology and Allergy Ankara Turkey; ^7^ Ankara City Hospital Clinic of Immunology and Allergic Diseases Ankara Turkey; ^8^ Istanbul University‐Cerrahpaşa Cerrahpaşa Faculty of Medicine Department of Pulmonary Diseases Istanbul Turkey; ^9^ University of Health Sciences Sureyyapasa Chest Diseases and Thoracic Surgery Training and Research Hospital Department of Immunology and Allergy Istanbul Turkey; ^10^ Istanbul University Istanbul Faculty of Medicine Department of Internal Medicine Division of Immunology and Allergic Diseases Istanbul Turkey; ^11^ University of Health Sciences Kocaeli Derince Training and Research Hospital Clinic of Allergy and Immunology Kocaeli Turkey; ^12^ University of Health Sciences Dr. Suat Seren Chest Diseases and Surgery Training and Research Hospital Department of Chest Diseases Division of Allergy and Immunology Izmir Turkey; ^13^ Hacettepe University Faculty of Medicine Department of Chest Diseases Division of Allergy and Clinical Immunology Ankara Turkey; ^14^ Diyarbakir Gazi Yasargil Training and Research Hospital Clinic of Immunology and Allergic Diseases Diyarbakır Turkey; ^15^ Kirikkale University Faculty of Medicine Department of Allergy and Immunology Kirikkale Turkey; ^16^ University of Health Sciences Yedikule Chest Diseases and Thoracic Surgery Training and Research Hospital Department of Chest Diseases Istanbul Turkey; ^17^ Mehmet Akif Inan Training and Research Hospital Clinic of Immunology and Allergy Diseases Sanliurfa Turkey; ^18^ Mersin University Faculty of Medicine Department of Chest Diseases Mersin Turkey; ^19^ Akdeniz University Faculty of Medicine Department of Chest Diseases Antalya Turkey; ^20^ Ege University Faculty of Medicine, Pulmonary, Immunology and Allergy Izmir Turkey; ^21^ Ordu University Training and Research Hospital Department of Pulmonology Ordu Turkey; ^22^ Ankara Yildirim Beyazit University Faculty of Medicine Ankara City Hospital Clinic of Immunology and Allergic Diseases Ankara Turkey; ^23^ Medicana International Ankara Hospital Division of Allergy and Immunology Ankara Turkey; ^24^ American Hospital Adult Allergy and Immunology Department Istanbul Turkey; ^25^ Kocaeli University Faculty of Medicine Department of Pulmonary Diseases Kocaeli Turkey; ^26^ Cyprus Internatıonal Unıversıty School of Medicine Department of Pulmonary Diseases Haspolat Cyprus; ^27^ Recep Tayyip Erdoğan University School of Medicine Department of Chest Diseases Rize Turkey; ^28^ Bursa City Hospital Clinic of Immunology and Allergic Diseases Bursa Turkey; ^29^ Gazi University School of Medicine Department of Pulmonary Diseases Ankara Turkey; ^30^ Ataturk University Faculty of Medicine Department of Pulmonary Disease Erzurum Turkey; ^31^ Baskent University Faculty of Medicine Department of Internal Medicine Division of Immunology and Allergy Ankara Turkey; ^32^ Aydin Adnan Menderes University School of Medicine Department of Pulmonology Aydin Turkey; ^33^ Bursa Uludag University Faculty of Medicine Department of Chest Diseases Division of Immunology and Allergy Bursa Turkey; ^34^ Adult Allergy Service Glenfield Hospital University Hospitals of Leicester Leicester UK; ^35^ Ege University Faculty of Medicine Department of Pulmonology Izmir Turkey; ^36^ Mersin City Training and Research Hospital Clinic of Chest Diseases Mersin Turkey; ^37^ Ondokuz Mayis University Department of Pulmonary Medicine Samsun Turkey; ^38^ Manisa Celal Bayar University Faculty of Medicine Department of Pulmonology Manisa Turkey; ^39^ Recep Tayyip Erdogan University School of Medicine Department of Chest Diseases Division of Allergy and Immunology Rize Turkey; ^40^ Biruni University School of Medicine Department of Chest Diseases Istanbul Turkey; ^41^ Lokman Hekim University Medical School Department of Respiratory Medicine Ankara Turkey; ^42^ Izmir Katip Celebi University Ataturk Training and Research Hospital Clinic of Immunology and Allergic Diseases Izmir Turkey; ^43^ Medeniyet University Faculty of Medicine Department of Allergy and Immunology Istanbul Turkey; ^44^ Dokuz Eylul University School of Medicine Department of Respiratory Diseases Izmir Turkey; ^45^ Private Immunology and Allergy Clinic Istanbul Turkey; ^46^ Van Regional Training and Research Hospital Clinic of Immunology and Allergic Diseases Van Turkey; ^47^ Hacettepe University School of Medicine Department of Ear Nose and Throat Ankara Turkey; ^48^ Ankara University School of Medicine Department of Otolaryngology‐Head and Neck Surgery Ankara Turkey; ^49^ University of Health Sciences Etlik City Hospital Department of Otolaryngology Ankara Turkey; ^50^ Ahmet Emre Suslu Private Ear Nose and Throat Clinic Ankara Turkey; ^51^ Lokman Hekim University Faculty of Medicine Department of Otorhinolaryngology Ankara Turkey

**Keywords:** asthma, asthma control, education level, household income, illiteracy, national database, social markers, socioeconomic status

## Abstract

**Background:**

Asthma is one of the most common causes of chronic respiratory disease, and countries with low socioeconomic status have both a high prevalence of asthma and asthma‐related death.

**Objective:**

In this study, we aimed to determine socioeconomic levels of asthmatic patients according to a national database and investigate the effects of social markers on disease control in our region.

**Methods:**

This is an analysis of data from 2053 adult asthma patients from a multicentre chart study in Turkey. Socioeconomic status (SES) data were collected from questionnaires and this form was sent to the patients via e‐mail. Parameters related to social status and poor disease control were analyzed.

**Results:**

Illiteracy (OR:2.687 [95% CI: 1.235–5.848]; *p* = 0.013) and lower household income (OR:1,76 [95% CI: 1.002–3.09]; *p* = 0.049) were found as independent risk factors for hospitalization in the multivariate logistic regression analysis. Therewithal, being aged between 40 and 60 (OR: 1.435 [95% CI: 1.074–1.917]; *p* = 0.015), illiteracy (OR: 2.188 [95% CI: 1.262–3.795]; *p* = 0.005) and being employed (OR: 1.466 [95% CI: 1.085–1.847]; *p* = 0.011) were considered as independent risk factors for systemic corticosteroid use at least 3 days within last 1 year.

**Conclusion:**

As a result of our national database, education level, household income and working status briefly socioeconomic status have impacts on asthma control. Identification of social markers in asthma and better recognition of risk factors based on the population gives us clues to provide better asthma control in the future.

## 1 INTRODUCTION

Asthma, a chronic respiratory disease, affects approximately 1%–18% of the population worldwide.^1^ According to the Global Burden of Disease, 495,100 individuals died of asthma in 2017 worldwide.^2^ Although asthma is more prevalent in high‐income countries, the majority of asthma‐related deaths occur in low‐ and middle‐income countries. Global efforts aimed at improving asthma diagnosis and treatment have not benefitted the most vulnerable communities. Significant challenges related to accurate diagnosis, the availability of effective treatments, and access to specialized care exist in most regions of the world.^3^

Socioeconomic status (SES) is an ambiguous term, and its scope has been determined differently across studies. It is the individual's position on the socioeconomic scale, determined by a combination of social and economic factors such as education level, household income, and insurance status.^4^ SES plays a significant role in determining health and nutritional status along with mortality and morbidity. Asthma prevalence tends to be higher in lower socioeconomic groups, particularly those with lower disease control status, than that in higher socioeconomic groups.^5^, ^6^ Lower SES individuals may be more exposed to environmental triggers such as pollution, tobacco smoke, and allergens owing to living in suboptimal housing conditions or neighborhoods with poor air quality than those from higher SES.^7^ Individuals with lower income were more likely to experience adverse asthma outcomes independent of education, perceived stress, race, and medication adherence^8^ Additionally, there is a substantial correlation between low levels of education and limited asthma knowledge. Ineffective inhaler use, adherence challenges, and limited knowledge regarding asthma action plans and triggers make asthma management difficult in these patients.^9^ Consequently, groups with low SES have higher rates of poor asthma control, hospital admissions, severe diseases, and asthma‐related deaths than those in groups with high SES.^10^, ^11^

Even though several studies have previously investigated the impact of SES on the health outcomes associated with asthma, these varied widely in their study populations, regions, healthcare systems, and methodologies. Studies aimed to assess the effects of SES on asthma in Turkish patients are scarce and conducted on a limited number of patients.^12^, ^13^ Having detailed information regarding the national asthma characteristics can offer treatment opportunities with a social integrative approach, and can guide in both disease management and risk stratification. Thus, this multicenter study aimed to determine the socioeconomic levels of patients with asthma according to a national registry and investigate the effects of social markers on disease control in Turkey.

## 2 METHODS

### 2.1 Turkish adult asthma registry

This is an analysis of data from a multicenter chart study and presents findings from a national registry on adult asthma, with entries recorded between March 2018 and March 2022 in Turkey. After the establishment of the idea of the registry, first, an invitation letter prepared by the study coordinator was e‐mailed to all members of the Asthma Working Group of the Turkish Thoracic Society. The requirement of being a study center was either secondary or tertiary hospitals with regular follow‐up patients with asthma. Finally, a total of 36 centers participated, and data from 2053 patients were recorded in the registry. SES data were collected from questionnaires standardized by the asthma database working group.^14^

### 2.2 Study patients

The study included patients aged 18 years and above, with confirmed asthma diagnoses based on both the Global Initiative for Asthma (GINA) and a minimum of 1 year of follow‐up, and provided informed consent. The study was approved by the Ankara University Ethics Committee (Approval no: 16–10 l I‐I7/2017).

#### 2.2.1 Study parameters and definitions

All parameters were collected from the national asthma database study.^14^ The study variables and outcomes were defined in a standardized manner based on guidelines by the study executive committee, and written instructions were mailed to all study investigators before data enrollment to the registry. The investigators were asked to enter the data in the registry. Non‐standardized data were neither entered nor accepted. Patient records were utilized as the data source. The database was used to collect the following information: *Parameters related to social status:* age, gender, place of birth (urban‐rural), place of residence (urban‐rural), marital and employment status, household income, education level, smoking history, and body mass index (BMI). *Parameters related to poor disease control:* parameters related to disease activity (exacerbation and hospitalization rates, and systemic steroid requirement related to asthma in the last year); and GINA asthma control category.

Asthma control was evaluated based on both the GINA assessment and asthma control test (ACT) categories.^1^, ^15^, ^16^ ACT is a validated patient‐reported asthma control measure consisting of five questions that assess activity limitation, shortness of breath, night‐time symptoms, use of rescue medications, and the patient's overall assessment of asthma control during the past 4 weeks. The sum of the responses to the questions is the ACT score, graded from 1 (worst) to 5 (best). The best score on the ACT is 25, and the worst is 5. Scores of 20–25, 16–19, and 5–15 are classified as well‐controlled, not well‐controlled, and very poorly controlled asthma, respectively.^15^, ^16^ The GINA symptom control tool (categorical classification) assesses the frequency of daytime symptoms, any night waking due to asthma or activity limitation, and frequency of the use of short‐acting beta agonist (SABA) reliever for symptom relief in patients in the past 4 weeks. Categorical classification ranges from 0 to 4, 0 indicating well‐controlled asthma, 1–2 partly controlled asthma, and 3–4 uncontrolled asthma.^1^

Combined household income level was collected as “Minimum wage and below,” “Between 2 and 4 times the minimum wage” and “>4 times the minimum wage.” The minimum wage is defined as “the lowest income necessary to meet a typical family's basic needs.” For univariate and multivariate analyses, education level was grouped as “illiterate,” “8 years of education and below” and “over 8 years of education.”

### 2.3 Statistical analysis

Data were described using either frequency (percentage) for categorical data or mean ± standard deviation (minimum‐maximum) for metric variables. Assumptions of normality and homogeneity of variances are assessed using the Kolmogorov–Smirnov and Levene tests, respectively. When comparing numerical variables, a Student *t*‐test was used to compare two groups, while group comparisons were conducted using Pearson's chi‐squared analysis for categorical variables.

Univariate and multivariable logistic regression analyses were used to define the risk factors for patients who used and did not use systemic corticosteroids for at least 3 days, those with and without hospitalization, and those with and without intensive care admission. The *p*‐value for inclusion in the multiple regression model was set at 0.20. Statistically significant value was set as *p* < 0.05. The IBM SPSS 22.0 software package (SPSS Inc., Chicago, IL, USA) was used to conduct the statistical analysis.

## 3 RESULTS

### 3.1 Patients' characteristics and disease control status

Table 1 presents the baseline characteristics of 2053 patients. Among them, the majority (*n* = 1535; 74.8%) were women. The mean age of the patients was 46.7 ± 14.7 years, with approximately 50% belonging to the 40–60 years age group (*n* = 1020; 50.1%).

**TABLE 1:** Demographics and socioeconomic status (SES) of the patients included in the study.

Variables	N
Gender; *n* (%)	
Female	1535 (74.8)
Male	518 (25.2)
Age; mean year ±SD	46.7 ± 14.7
Age groups; *n* (%)	
18–39 years	639 (31.4)
40–60 years	1020 (50.1)
≥60 years	375 (18.4)
Marital status; *n* (%)	
Married	778 (37.9)
Single	223 (10.9)
Divorced	42 (2)
Level of education; *n* (%)	
Illiterate	83 (4.3)
No school, literate	30 (1.6)
Primary school	766 (39.9)
High school	451 (23.5)
Collage/University	588 (30.7)
Employment; *n* (%)	
Government officer	279 (14.4)
Self‐employed	256 (13.2)
Housewife	795 (41)
Student	147 (7.6)
Retired	189 (9.7)
Unemployed	93 (4.8)
Body mass index; mean ± SD	28.1 ± 5.6
Body mass index groups; *n* (%)	
≤18.49 (weak)	38 (1.9)
18.50–24.99 (normal)	569 (27.7)
25–29.99 (overweight)	693 (33.8)
30–34.99 (1st degree obese)	407 (19.8)
35–39.99 (2nd‐degree obese)	145 (7.1)
≥40 (3rd degree obese)	67 (3.3)
Smoking history; *n* (%)	
Never smoked	1364 (68,1)
Currently smoker	228 (11.4)
Ex‐smoker	410 (20.5)
Cigarette; mean package/years ±SD	14 ± 22.2
Place of birth; *n* (%)	
Urban	1136 (55.3)
Rural	800 (39)
Place of residence; *n* (%)	
Urban	1854 (90.3)
Rural	117 (5.7)
Household income; *n* (%)	
Minimum wage and below	386 (21.8)
Between 2 and 4 times the minimum wage	846 (41.2)
>4 times the minimum wage	540 (26.3)

Abbreviation: SD, standard deviation.

### 3.2 SES outcomes of the study group

The majority of the patients included were unemployed, 41% (*n* = 795) were housewives, and the highest grade of education in 40% (*n* = 766) was a primary school. The vast majority of patients were born and residents of an urban area (*n* = 1136 [55.3%] and *n* = 1854 [90.3%], respectively). The household income of the participants was low and mostly 2–4 times the minimum living wage (*n* = 560; 61.7%) (Table 1). The majority of the patients were using ICS/LABA combinations (*n* = 1811, 88%) and on GINA step 3–5 treatments [*n* = 443 (25.6%), *n* = 388 (22.4%), and *n* = 694 (40.1%).

### 3.3 Asthma control status

Approximately one‐third of the patients (*n* = 671, 32.6%) were diagnosed with severe asthma, while 934 participants (46%) exhibited well‐controlled asthma based on GINA assessment. Regarding disease activity in the past year, 25% (*n* = 431), 27.3% (*n* = 482), 8.7% (*n* = 150), and 1.2% (*n* = 20) had a history of exacerbations requiring at least 3 days of systemic corticosteroid use, a history of emergency service admission, hospitalized due to asthma, and were followed up in an intensive care unit (ICU) within the last year, respectively (Table 2). Of the 171 patients (41.5%) who visited the emergency room once owing to asthma, 71 had severe asthma, and 184 of the 311 patients (58.2%) who visited the emergency room twice or more in the previous year had severe asthma.

**TABLE 2:** Clinical characteristics of the included patients.

Variable	N
Disease duration; mean years ±SD	12.9 ± 10.34
Presence of severe asthma; *n* (%)	670 (32.6)
Asthma control, *n* (%) (GINA assessment)	
Well controlled	934 (46.4)
Partial controlled	610 (30.3)
Uncontrolled	468 (23.3)
Exacerbations requiring at least 3 days of use of OCS in the last year; *n* (%)	431 (24.5)
Presence of emergency service admission due to asthma in the last 1 year; *n* (%)	482 (27.3)
Presence of hospitalization due to asthma in the last 1 year; *n* (%)	150 (8.7)
Presence of intensive care hospitalization due to asthma in the last 1 year; *n* (%)	20 (1.2)

### 3.4 SES factors and their associations with asthma control

Household income and level of education directly affect asthma control and with their increase, the percentage of patients with well‐controlled asthma increases (Table 3).

**TABLE 3:** Effects of household income and education level on the asthma control test (ACT) scores.

Asthma control test scores				
	5–15	16–19	20–25	*p*
Household income				
Minimum wage and below	84 (26.2)	64 (19.9)	173 (53.9)	<0.001
Between 2 and 4 times the minimum wage	146 (20.6)	170 (24)	392 (55.4)	
>4 times the minimum wage	75 (16.6)	78 (17.3)	299 (66.2)	
Level of education				
Illiterate	24 (35.3)	12 (17.6)	32 (47.1)	**0.007**
8 years of education and below	146 (21.9)	141 (21.2)	379 (56.9)	
Over 8 years	155 (18.2)	168 (19.7)	528 (62)	

*Note:* Bold values denote statistical significance at the *p* < 0.05 level.

### 3.5 Age, level of education, and employment status affect the need for using systemic corticosteroids

Based on their education levels, age groups, and working status, differences were observed among patients who required systemic steroids for at least 3 days within the last year and those who did not (Table 4). The former group was older and had a lower level of education and a higher rate of employment.

**TABLE 4:** General characteristics of patients who used and did not use steroids for asthma for at least 3 days.

Variable	With systemic corticosteroid use for at least 3 days	No systemic corticosteroid use	*p*
Female gender; *n* (%)	327 (76)	994 (75)	0.653
Age; mean years ±SD	48.3 ± 13.5	46.8 ± 14.8	0.054
Age group; *n* (%)			
18–39	110 (25.8)	418 (31.7)	**0.028**
40–65	240 (56.2)	648 (49.2)	
≥65	77 (18)	252 (19.1)	
Place of residence (rural/Urban)	25/391	77/1196	0.977
Level of education; *n* (%)			
Illiterate	26 (6.4)	43 (3.4)	**0.028**
8 years and below	175 (43.3)	545 (43.4)	
over 8 years	203 (23.3)	667 (53.1)	
Occupation; *n* (%)			
Employee (officer, self‐employed other)	165 (40.4)	434 (34.1)	**0.021**
Unemployed (student, retired, housewife, or unemployed)	243 (59.6)	837 (65.9)	
BMI; *n* (%)			
<30	261 (64.9)	862 (67.7)	0.3
≥30 (obese)	141 (35.1)	411 (32.3)	
BMI; mean ± SD	28.5 ± 5.9	28.1 ± 5.5	0.156
Smoking history; *n* (%)			
Never smoked	39	156	0.066
Currently smoker	102	258	
Ex‐smoker	278	898	
Cigarette (package/year) (mean ± SD) (median, min‐max)	13.9 ± 21.4	14.3 ± 23.6	0.857
Household income			
Minimum wage and below	48 (25.1)	153 (27)	0.594
Between 2 and 4 times the minimum wage	113 (59.2)	340 (60.1)	
>4 times the minimum wage	30 (15.7)	73 (12.9)	

*Note:* Bold values denote statistical significance at the *p* < 0.05 level.

In multivariate logistic regression analysis, age between 40 and 60 years (odds ratio [OR]: 1.435 [95% confidence interval [CI]: 1.074–1.917]; *p* = 0.015), illiteracy (OR: 2.188 [95% CI: 1.262–3.795]; *p* = 0.005), and employment (OR: 1.466 [95% CI: 1.085–1.847]; *p* = 0.011) were considered as independent risk factors for systemic corticosteroid use at least 3 days within last 1 year (see eTable 1).

### 3.6 SES factors associated with hospitalization in the last year owing to asthma

Within the last year, 150 patients (8.7%) were hospitalized owing to asthma exacerbation. These patients had a higher BMI (29.4 ± 6.6 vs. 28.1 ± 5.5; *p* = 0.021), were more likely to be born in rural areas (49.6% vs. 40.9%; *p* = 0.047), and had lower household income when compared with those without hospitalization (Table 5).

**TABLE 5:** General characteristics of patients with and without hospitalization.

Variable	With hospitalization	Without hospitalization	*p*
Gender, female; *n* (%)	119 (79.3)	1172 (74.6)	0.197
Age; mean years ±SD	47.08 ± 13.9	47.2 ± 14.5	0.934
Age group; *n* (%)			
18–39	44 (29.3)	471 (30.2)	0.934
40–65	79 (52.7)	796 (51.1)	
≥65	27 (18)	291 (18.7)	
Level of education; *n* (%)			
Illiterate	13 (9.2)	53 (3.6)	**0.001**
8 years and below	69 (48.6)	639 (43.2)	
Over 8 years	60 (42.3)	787 (53.2)	
Occupation; *n* (%)		
Employee (officer, self‐employed other)	50 (34.2)	535 (35.7)	0.732
Unemployed (student, retired, housewife, or unemployed)	96 (65.8)	965 (64.3)	
BMI; *n* (%)			
<30	84 (60.4)	1015 (67.7)	0.081
≥30 (obese)	55 (39.6)	484 (32.3)	
BMI; mean ± SD	29.4 ± 6.6	28.1 ± 5.5	**0.021**
Number of people in the household median (min‐max)	3 (1–10)	3 (1–11)	0.254
Smoking status; *n* (%)			
Current smoker	17	176	0.357
Ex‐smoker	38	324	
Never smoked	92	1049	
Place of birth (urban/rural)	70/69	881/611	**0.047**
Place of residence (urban/rural)	132/14	1420/87	0.066
Household income; *n* (%)			
Minimum wage and below	19 (34.5)	180 (26.7)	0.375
Between 2 and 4 times the minimum wage	28 (50.9)	406 (60.1)	
>4 times the minimum wage	8 (14.5)	89 (13.2)	
Household income; *n* (%)			**0.005**
Under 1000 TL	9 (6.9)	112 (8.1)	
1000–2000 TL	32 (24.6)	187 (13.6)	
2000–5000 TL	60 (46.2)	654 (47.6)	
Over 5000 TL	29 (22.3)	422 (30.7)	

*Note:* Bold values denote statistical significance at the *p* < 0.05 level.

In the multivariate logistic regression analysis, only illiteracy (OR: 2.687 [95% CI: 1.235–5.848]; *p* = 0.013) and lower household income (OR: 1,76 [95% CI: 1.002–3.09]; *p* = 0.049) were found as independent risk factors for hospitalization (see eTable 2).

### 3.7 Differences in education level, place of birth, and place of residence in patients followed up at ICUs

Only a small number of patients required ICU support due to asthma exacerbation (*n* = 20, 1.2%). Noteworthy differences were observed in education levels, place of birth, and place of residence between patients requiring ICU support for asthma and those who did not. In univariate analyses, the rate of intensive care hospitalization was higher in those who were illiterate (OR: 10,64 [95% CI: 2.327–45.65]; *p* = 0.002) and with less than 8 years of education (OR: 3666 [95% CI: 1.177–11.41]; *p* = 0.025) than in others. Additionally, it was more common for patients born in rural areas (OR: 4.29 [95% CI: 1.554–11.884]; *p* = 0.005) and those still residing in urban areas (OR: 7.081 [95% CI: 1.554–11.884]; *p* < 0.001) in univariate analyses. However, multivariate analysis could not be performed owing to statistical limitations.

## 4 DISCUSSION

This was one of the most comprehensive studies involving a substantial number of Turkish adult patients with asthma. This study's results demonstrate the significant impact of social factors, particularly education level and household income, on asthma control in terms of the ACT score, hospitalization rates, and the need for systemic corticosteroids. We observed that illiteracy is a risk factor for both the use of systemic steroids for at least 3 days and hospitalization; moreover, low household income is also a risk factor for hospitalization. It was also observed that better asthma control was achieved as education level and household income increased.

When the social profile of Turkish patients with asthma was evaluated, the education level demonstrated heterogeneous distribution; however, the most common education level was primary school, and the majority of patients were living in urban areas with a BMI above normal. The majority of patients were concentrated in middle and low‐income levels (minimum wage and below, between 2 and 4 times the minimum wage) and were not working. Similarly, in another Turkish study, the majority lived in urban areas, with the most common occupation being housewife.^13^ In a study conducted in the American population, patients with asthma had a high school or higher level of education (84.6%), lived in rural areas, and had heterogeneous income levels.^17^ The differences between Turkish and American societies show that each society has its own characteristics.

We observed a positive correlation between the ACT score and education and household income levels. When examined on a regional basis, the number of patients with poor control was higher in the South‐eastern and Eastern Anatolia regions, which were regions with low literacy rates. In accordance with our results, another study found higher levels of education and federal poverty to be positively correlated with improved mean ACT scores.^18^ When the data of elderly female patients in France were examined, women with low SES had more often uncontrolled asthma.^19^ Azeez et al. showed that monthly income is the only predictor of good asthma control.^20^ In another study, including African–American/Black and Hispanic/Latino populations, differences in ACT and Asthma Activities, Persistent, triGGers, Asthma medications, and Response to therapy (APGAR) were only observed at the education level when high school and below were compared with university and above.^21^ A low education level may lead to poor adherence to treatment and inadequate inhaler device‐using skills, resulting in poor asthma control.^9^ Limited knowledge of asthma management strategies, triggers, and symptoms contributes to this challenge. As income level increases, health accessibility increases, alleviating financial concerns. Having a higher household income was shown to be the only socioeconomic factor associated with better asthma control.^12^ A study conducted in a Western Norwegian community demonstrated an increased frequency of asthma and respiratory symptoms among individuals with lower educational levels.^5^ Additionally, increased asthma prevalence was associated with low educational levels^22^; however, allergic diseases and asthma with atopy were associated with higher SES in other studies.^6^, ^23^

The factors underlying poor asthma control in individuals with low income are unknown, although there are studies suggesting that nutritional deficiencies might be a factor associated with poor asthma control^24^, ^25^ Simultaneously, one of the elements that contribute to inadequate asthma control might be insufficient access to healthcare services and the inability to afford treatment costs. The use of an inhaler corticosteroid (ICS)‐containing controller to improve symptom control, and lower the risk of exacerbations and asthma fatalities is essential in asthma management.^1^ Low‐ and middle‐income countries have highly limited access to or cannot afford ICS‐containing inhalers.^26^ Another contributing factor in low and middle‐income countries may be the use of biomass fuel for cooking, lighting, and heating, predisposing them to frequent acute asthma exacerbation and recurrent chest infections^27^; however, these were not assessed in this study.

In most studies on SES, the patient's health insurance status was also determined. The absence of health insurance is indirectly associated with low income. Patients without health insurance were found less likely to receive regular asthma care, such as lower use of ICS (41%, 41%, and 29%; *p* < 0.001) and asthma specialist care (9%, 10%, and 4%; *p* < 0.001) that was recommended by the guidelines, even when their disease severity was higher.^28^ A more recent study found that insurance coverage was associated with improved asthma control in adults aged 18–64 years from low SES households.^29^ However, the use of private health insurance is highly limited in Turkey with only 0.7% of our patients having private health insurance; however, the insurance status of the patients was not evaluated in this study. Approximately 94% of them can access health services free of charge by the Social Insurance Institution. Covering health expenses by the social insurance institution in Turkey might further provide an advantage in terms of disease control.

Our results indicated that systemic corticosteroid use and hospitalization rates, which are undesirable consequences of impaired asthma control, were higher in illiterate patients. A recent nationwide study evaluating pediatric asthmatics of the Korean population also associated low SES with increased systemic steroid use and hospitalization rates.^30^ Additionally, we observed a higher need for systemic corticosteroid use in the employees, which may depend on the occupational exposures to both sensitizers and irritants associated with current adult‐onset asthma and uncontrolled asthma.^31^ The desire to shorten the exacerbation periods and prevent the reduction in the number of days worked may be other possible reasons for the higher use of steroids. Despite free healthcare services in Turkey, unemployed patients may not seek hospital care owing to financial concerns.

Limited access to foods, a sedentary lifestyle, smaller family size, the use of antibiotics, environmental pollution, and migration have all been identified as risk factors for asthma. These factors are related to environmental and lifestyle changes brought on by urbanization.^32^ Although multivariate analyses do not support this, we found higher rates of hospitalization and ICU admission in patients born in rural areas in univariate analyses and higher rates of ICU admission in residents in urban. Limited data show that rural patients generally have difficulties in accessing health services, which suggests that they receive inadequate care for asthma.^33^, ^34^ The availability of insurance was linked to higher rates of hospitalization for asthma in non‐rural areas; however, in rural areas, income, occupation, and the proportion of people who do not speak English as their first language were linked to lower rates in another study.^35^

All societies have different dynamics owing to geographical, ethnic, and financial differences; therefore, the results related to SES may not be compatible with the structure of each society. For instance, in Canada, lower SES was not related to poor asthma‐related quality of life.^36^ Inequalities within and even between communities have been repeatedly documented worldwide, including in countries with publicly supported health systems.^37^ Therefore, each society should be evaluated on its merits.

Since a large number of participants from many centers from every region of the country were included, we believe that the patient group objectively reflects the socioeconomic level of the Turkish adult patient population with asthma.

The retrospective study design limited our ability to evaluate other parameters reflecting SES. Additionally, it is important to note that asthma patients included in the study were part of a group with more hospital admissions due to uncontrolled asthma, which could potentially impact the results.

In conclusion, the national asthma database study provides valuable insights into the demographics, and phenotypic categories of asthma in the Turkish population, shedding light on the intricate relationship between SES and asthma control. Consequently, low SES can be considered a risk factor for asthma exacerbations and uncontrolled asthma. Identification of social markers in asthma and better recognition of risk factors based on the population gives us clues to provide better asthma control in the future. In light of these markers, specific populations can be identified, and tailored approaches to asthma management can be performed for these groups. A more significant disease burden means a higher cost of society. The findings of this study may raise awareness of these significant risk factors and provide information on potential avenues for preventing fatal or near‐fatal asthma. Social policies aiming to increase the education level of the population and to improve access to health care or to reduce environmental triggers should be prioritized.

## AUTHOR CONTRIBUTIONS

**Bahar Arslan**: Writing ‐ original draft; Writing ‐ review and editing; Investigation; Validation. **Murat Tuerk**: Writing ‐ original draft; Writing ‐ review and editing; Methodology; Data curation; Conceptualization; Investigation. **Serhat Hayme**: Data curation; Validation. **Omuer Aydin**: Conceptualization; Investigation; Methodology; Validation. **Derya Gokmen**: Data curation; Software. **Gozde Koycu Buhari**: Investigation; Methodology. **Zeynep Celebi Sozener**: Conceptualization; Investigation; Methodology. **Bilun Gemicioglu**: Supervision; Investigation; Methodology. **Ismet Bulut**: Investigation; Methodology. **Sengul Beyaz**: Investigation; Methodology. **Cihan Orcen**: Investigation; Validation. **Secil Kepil Ozdemir**: Investigation; Methodology. **Metin Keren**: Methodology; Investigation. **Ebru Damadoglu**: Investigation; Methodology. **Tugce Yakut**: Investigation; Methodology. **Ayse Fusun Kalpaklioglu**: Investigation; Methodology; Conceptualization. **Ayse Baccioglu**: Investigation; Methodology; Conceptualization. **Sumeyra Alan Yalim**: Investigation; Validation. **Insu Yilmaz**: Investigation; Validation; Methodology. **Ilkay Koca Kalkan**: Investigation; Methodology. **Elif Yelda Ozgun Niksarlioglu**: Investigation; Validation. **Ali Fuat Kalyoncu**: Conceptualization; Investigation; Writing ‐ original draft; Methodology. **Gul Karakaya**: Investigation; Methodology. **Muge Erbay**: Investigation; Methodology. **Sibel Nayci**: Investigation; Validation. **Fatma Merve Tepetam**: Investigation; Methodology. **Asli Akkor Gelincik**: Investigation; Validation; Methodology. **Hulya Dirol**: Investigation; Validation. **Ozlem Goksel**: Investigation; Validation. **Selen Karaoglanoglu**: Investigation; Validation. **Ferda Oner Erkekol**: Investigation; Validation. **Sacide Rana Isik**: Investigation; Validation. **Fusun Yildiz**: Investigation; Validation. **Yasemin Yavuz**: Investigation; Validation. **Dilek Karadogan**: Investigation; Validation. **Nurgul Bozkurt**: Investigation; Validation. **Ummuhan Seker**: Investigation; Validation. **Ipek Kivilcim Oguzulgen**: Investigation; Validation. **Ilknur Basyigit**: Investigation; Validation. **Serap Argun Baris**: Investigation; Validation. **Elif Yilmazel Ucar**: Investigation. **Tuba Erdogan**: Investigation; Validation. **Mehmet Polatli**: Investigation; Validation. **Dane Ediger**: Investigation; Validation. **Fatma Esra Gunaydin**: Investigation; Validation. **Leyla Pur**: Investigation; Validation. **Zeynep Yegin Katran**: Investigation; Validation. **Yonca Sekibag**: Investigation; Validation. **Enes Furkan Aykac**: Investigation; Validation. **Dilsad Mungan**: Conceptualization; Investigation; Methodology. **Ozcan Gul**: Investigation; Validation. **Ali Cengiz**: Investigation; Validation. **Bulent Akkurt**: Investigation; Validation. **Seyma Ozden**: Investigation; Validation. **Semra Demir**: Investigation; Validation. **Derya Unal**: Investigation; Validation. **Ayse Feyza Aslan**: Investigation; Validation. **Ali Can**: Investigation; Validation. **Reyhan Gumusburun**: Investigation. **Gulhan Bogatekin**: Validation; Investigation. **Hatice Serpil Akten**: Investigation; Validation. **Sinem Inan**: Investigation; Validation. **Munevver Erdinc**: Investigation; Validation. **Aliye Candan Ogus**: Investigation. **Murat Kavas**: Investigation. **Demet Polat Yulug**: Validation; Investigation. **Mehmet Erdem Cakmak**: Investigation. **Saltuk Bugra Kaya**: Investigation; Validation. **Gulistan Alpagat**: Investigation; Validation. **Eylem Sercan Ozgur**: Investigation; Validation. **Oguz Uzun**: Investigation; Validation. **Sule Tas Gulen**: Investigation; Validation. **Gulseren Pekbak**: Investigation; Validation. **Deniz Kizilirmak**: Investigation; Validation. **Yavuz Havlucu**: Investigation; Validation. **Halil Donmez**: Investigation; Validation. **Gulden Pacaci Cetin**: Validation; Investigation. **Sadan Soyyigit**: Investigation; Validation. **Bilge Yilmaz Kara**: Validation; Investigation. **Gulden Pasaoglu Karakis**: Investigation; Validation. **Adile Berna Dursun**: Investigation; Validation; Methodology. **Resat Kendirlinan**: Investigation; Validation. **Ayse Bilge Ozturk**: Investigation; Validation. **Can Sevinc**: Investigation; Validation. **Gokcen Omeroglu Simsek**: Investigation; Validation. **Oznur Abadoglu**: Investigation; Validation. **Pamir Cerci**: Investigation; Validation. **Taskin Yucel**: Investigation; Validation. **Irfan Yorulmaz**: Investigation; Validation. **Zahide Ciler Tezcaner**: Investigation; Validation. **Emel Cadalli Tatar**: Investigation; Validation. **Ahmet Emre Suslu**: Investigation; Validation. **Serdar Ozer**: Investigation; Validation. **Engin Dursun**: Investigation; Validation. **Arzu Yorgancioglu**: Conceptualization; Investigation; Validation; Methodology. **Gulfem Elif Celik**: Conceptualization; Investigation; Methodology; Visualization; Writing ‐ review and editing; Formal analysis; Supervision. **Mehmet Atilla Uysal**: Supervision; Investigation; Methodology.

## CONFLICT OF INTEREST STATEMENT

All authors declare that they do not have any potential conflicts of interest in relation to any aspect of this work.

## Supporting information

Supplementary Material
