# Supplementary material for: Socioeconomic status has direct impact on asthma control: Turkish adult asthma registry
Source: Clin Transl Allergy. 2025 Jan 25;15(1):e70018. doi: 10.1002/clt2.70018 (PMC11761715; doi:10.1002/clt2.70018)
Supplement: Supplementary file 1 — Supplementary Material [file CLT2-15-e70018-s001.docx]

**eTable 1.** Univariate and multivariate comparisons of patients who used and did not use systemic corticosteroids for a minimum period of 3 days.

|  | **Univariate** | | | **Multivariate** | | |
| --- | --- | --- | --- | --- | --- | --- |
|  | **OR** | **95%CI** | ***p*** | **OR** | **95%CI** | ***p*** |
| Age group  18–39  40–60  >60 | 1,000  1.407  1.161 | 1.088–1.820  0.834–1.616 | **0.009**  0.376 | 1,000  1.435  1.214 | 1.074–1.917  0.828–1.781 | **0.015**  0.32 |
| Level of education  Illiterate  8 years of education and below  8 years of education and above | 1.987  1.055  1,000 | 1.191–3.314  0.837–1.33 | **0.009**  0.65 | 2.188  1.083  1,000 | 1.262–3.795  0.830–1.414 | **0.005**  0.556 |
| Employment status  Employee  Unemployed | 1.31  1,000 | 1.041–1.647 | **0.021** | 1.416  1,000 | 1.085–1.847 | **0.011** |
| Smoking status  Never smoked  Ex-smoker  Current smoker | 1,000  1.277  0.808 | 0.979–1.666  0.555–1.176 | 0.071  0.265 | 1,000  1.259  0.831 | 0.953–1.664  0.558–1.238 | 0.104  0.363 |
| BMI  <30  ≥30 (obese) | 1.000  1.133 | 0.895–1.435 | 0.3 | - | - | - |

OR*:* Odds Ratio

**eTable 2.** Univariate and multivariate comparisons of patients with and without hospitalization.

|  | **Univariate** | | | **Multivariate** | | |
| --- | --- | --- | --- | --- | --- | --- |
|  | **OR** | **95%CI** | **p** | **OR*** | **95%CI** | **P** |
| Gender  Male  Female | 1.000  1.31 | 0.868–1.977 | 0.198 | 0.969 | 0.598–1.57 | 0.898 |
| Level of education  Illiterate  8 years of education and below  8 years of education and above | 3.217  1.416  1.000 | 1.661–6.321  0.987–2.033 | **0.001**  0.059 | 2.687  1.331 | 1.235–5.848  0.835–2.122 | **0.013**  0.229 |
| Place of birth  Urban  Rural | 0.704  1.000 | 0.497–0.997 | **0.048** | 0.844 | 0.553–1.29 | 0.434 |
| Place of residence  Urban  Rural | 0.578  1.000 | 0.32–1.044 | 0.069 | 0.752 | 0.382–1.481 | 0.41 |
| BMI, n (%)  *<30*  *≥30 (obese)* | 1.000  1.373 | 0.961–1.962 | 0.082 | 1.2 | 0.795–1.827 | 0.378 |
| Monthly income  Minimum wage and below  Up to 2 times the minimum wage  Minimum wage >2 times | 1.995  1.335  1.000 | 1.213–3.284  0.843–2.114 | **0.007**  0.218 | 1.76  1.204 | 1.002–3.09  0.717–2.023 | **0.049**  0.483 |
